# We are the Earthworms! Aliens Using 3R on Humans: A Qualitative Experimental Ethics Study with Animal Research Professionals

**DOI:** 10.1007/s10806-026-09973-9

**Published:** 2026-02-17

**Authors:** Christian Rodriguez Perez, Kirsten Persson, David M. Shaw

**Affiliations:** 1 Institute for Biomedical Ethics, University of Basel, Basel, Switzerland; 2 Institute for Animal Hygiene, Animal Welfare and Farm Animal Behaviour, University of Veterinary Medicine Hannover, Hannover, Germany; 3 Care and Public Health Research Institute Maastricht University, Maastricht, Netherlands

**Keywords:** 3R principles, Experimental philosophy, Speciesism, Animal research, Aliens, Thought experiment

## Abstract

This study explores animal research professionals’ attitudes toward the 3R principles (i.e., 3R: replacement, reduction, and refinement of the use of animals in research) using an experimental ethics approach. A thought experiment involving a superior extraterrestrial alien species conducting research on humans according to 3R was presented to 13 Swiss-based animal research professionals (i.e., researchers using animals, veterinarians, animal welfare officers, 3R coordinators, animal science trainers, and members of animal experimentation commissions) during qualitative semi-structured interviews. Participants were asked to reflect on whether the aliens’ research is justified, as well as on the extent to which 3R is the right framework for it. Thematic analysis was used to analyze the data. Participants revealed a spectrum of attitudes, ranging from ambivalent acceptance to outright rejection of “3R for humans”. Key themes included concerns about legitimacy, consent, and power asymmetry, as well as anthropocentric and speciesist attitudes. These findings support previously identified concerns about the limitations of 3R and show that professionals also recognize these challenges when reflecting on the issue using a role-reversal thought experiment. This underscores the ongoing need to advance ethical frameworks in animal research, expanding beyond 3R to incorporate broader moral considerations. Ultimately, our findings suggest that empirical animal ethics, and in particular thought experiments of this kind, offers a promising route to fostering ethical reflection and reform.

## Introduction

What if extraterrestrial aliens applied the same principles to research on humans that humans apply to research on non-human animals (henceforth “animals”)?

The 3R principles (i.e., 3R—replacement, reduction, and refinement of the use of animals in research) play an important role in justifying the use of animals in research and are increasingly implemented in legislation worldwide (Grimm et al., 2023; Bayne et al., 2015; World Animal Protection, 2025). These principles aim to ensure that the harm caused to animals in research is minimized to the necessary amount, meaning that the benefits of research could not be achieved by causing less harm (Tannenbaum & Bennett, 2015). Previous research suggests that animal research professionals hold both positive and negative views on 3R (Blomberg, 2019). But would their attitudes to 3R shift if, by analogy, a superior alien species (more powerful, and hyperintelligent) applied 3R to their research on humans?

In this paper, we explore this question using a qualitative experimental ethics approach. Experimental ethics intends to answer moral questions relying primarily on experiments (Knobe et al., 2012; Pölzler & Paulo, 2021). In this context, thought experiments are key tools to access ethically relevant information through mere reflection (Pölzler & Paulo, 2021). To challenge people’s intuitions, thought experiments tend to be deprived of influential contextual factors of everyday moral decisions (Persson, 2018).

With this in mind, we created the “Superior Scientist” thought experiment which we used as the last part of 13 semi-structured interviews with animal research professionals. Our goal was not to provide a definitive normative evaluation of 3R, but rather to challenge intuitions and investigate attitudes on the role of 3R in animal research. In addition to contributing to the literature regarding the ethics of human-animal relationships, this study offers an innovative empirical animal ethics approach.

## Background

### The Role of 3R in Animal Research

The 3R principles are widely endorsed in animal research guidelines and increasingly reflected in legislation around the globe (Grimm et al., 2023; Bayne et al., 2015; World Animal Protection, 2025). While definitions of the three Rs vary, they can be described as aiming to minimize the harms to animals used in research (Rodriguez Perez et al., 2023; Louis-Maerten et al., 2024), without challenging the legitimacy of their use (Müller, 2024).

As this study is part of a Swiss National Research Program and involves Swiss-based experts, let us briefly consider the case of Switzerland. In Switzerland, animal research is legally justified if it is demonstrated to be “indispensable” (SR 455, Art. 17). The research is considered indispensable if, and only if, it is suitable (i.e., scientifically valid), necessary (i.e., harms have been minimized according to 3R), it pursues a legitimate societal interest (i.e., protection of the life and health of humans and animals, or of the natural environment, or production of new knowledge on fundamental processes of life) and the expected benefits outweigh the harms to the animals (Swiss Academies of Arts and Sciences, 2022). In this context, applying 3R is required to comply with the necessity criterion (SR 455.1, Art. 137 (2) and (4)(a).

Legal justification does not equal moral justification. The difference between the two in this case, however, is not straightforward. The Swiss legal justification process described above is supposed to balance constitutional goods. On the one hand, the Swiss constitution recognizes that human and animal life and health should be protected (SR 101, Art. 78, 80, 118), just like the environment (SR 101, Art. 74, 78). On the other hand, it also recognizes that animals have dignity (SR 101, Art. 120) and that their dignity, including their wellbeing, should be protected (SR 101, Art. 80; SR 455, Art. 3). We can see how value judgements and norms are involved in these considerations. It is outwith the scope of this paper to resolve this tension between legal and moral justification in this specific case, and we will thus consider both when investigating participants’ attitudes.

The justification of human research (by humans) has different requirements from the justification of animal research (by humans). Among other reasons, one is that where human dignity is considered absolute, animal dignity can be considered relative and readily overridden by competing interests (Shaw et al., 2024; Persson et al., 2017).

### The Alien Analogy: Aim and Hypothesis

The idea that extraterrestrial aliens may have superior capacities compared to humans has previously been used in animal ethics thought experiments. One example is Firestone’s (2016) thought experiment which asks us to imagine that aliens arrive and start treating humans in the same way they treat animals (e.g., confining them in farms to produce human meat, doing experiments on them). Just as humans control other species, aliens can be imagined to control humans. Placing aliens as the “superior species” in this way enables us to challenge our intuitions on the ethics of human-animal relationships and to deprive scenarios of influential contextual factors of everyday moral situations (e.g., in this case, human rights or informed consent) (Persson, 2018; Schneeberger, 2022). Importantly for ethical analysis, this type of role-reversal thought experiment asks participants to assess moral rules from a different positional perspective, testing impartiality and consistency in line with the Golden Rule, a strategy particularly fruitful for revealing biases and encouraging deeper ethical reflection (Pölzler & Paulo, 2021). What makes our approach novel is combining such framework with qualitative research from the perspective of experts in the field of animal research.

As stated, our goal with the Superior Scientist thought experiment was not to provide a definitive normative evaluation of 3R, but rather to perform an exploratory study of participants’ attitudes. To guide our conception and analysis, we used concepts from animal and human research ethics and law. We intentionally framed our thought experiment to make the “alien-human, human-animal” analogy as clear as possible. We relied on common components of animal research justification discussed above, such as suitability, legitimacy, and definitions of dignity. Additionally, we considered the relevance of species-specific characteristics (i.e., extraterrestrial, human, non-human), the potential for anthropocentrism and speciesism (i.e., the assignment of different moral worth based on species membership) in participants’ attitudes (Caviola et al., 2019), and the general criticisms often made of 3R (Louis-Maerten et al., 2024).

We formed exploratory hypotheses to guide our analysis. In doing so, we considered that our attitudes towards humans and nonhuman animals are shaped by several assumptions, some of them more and some less morally relevant. Also, that we are prone to cognitive bias, for example when evaluating different species’ mental capacities (Caviola & Capraro, 2020). In this regard, we might adhere to contradictory or unjustified beliefs. Moreover, people often try to justify their initial gut feelings through rationalization (Haidt, 2012). That could be the case for those who are dealing with such beliefs on a daily basis. We expected some participants to accept the analogy, others to reject it, and some to resist it with less certainty. We anticipated that participants could have a gut feeling that applying 3R in human research is wrong and search for reasons to support the claim, reflecting the common view that human research ethics requires autonomy and consent (Beauchamp & Childress, 2019). We expected the difference between aliens, humans, and other animals, for example in terms of cognitive capacities, to play a role in participants’ attitudes. Finally, we asked for alternative frameworks to guide the aliens’ research (i.e. we were open to the idea that the participants would come up with further criteria).

## Methods

### The Superior Scientist Thought Experiment

**Fig. 1:**
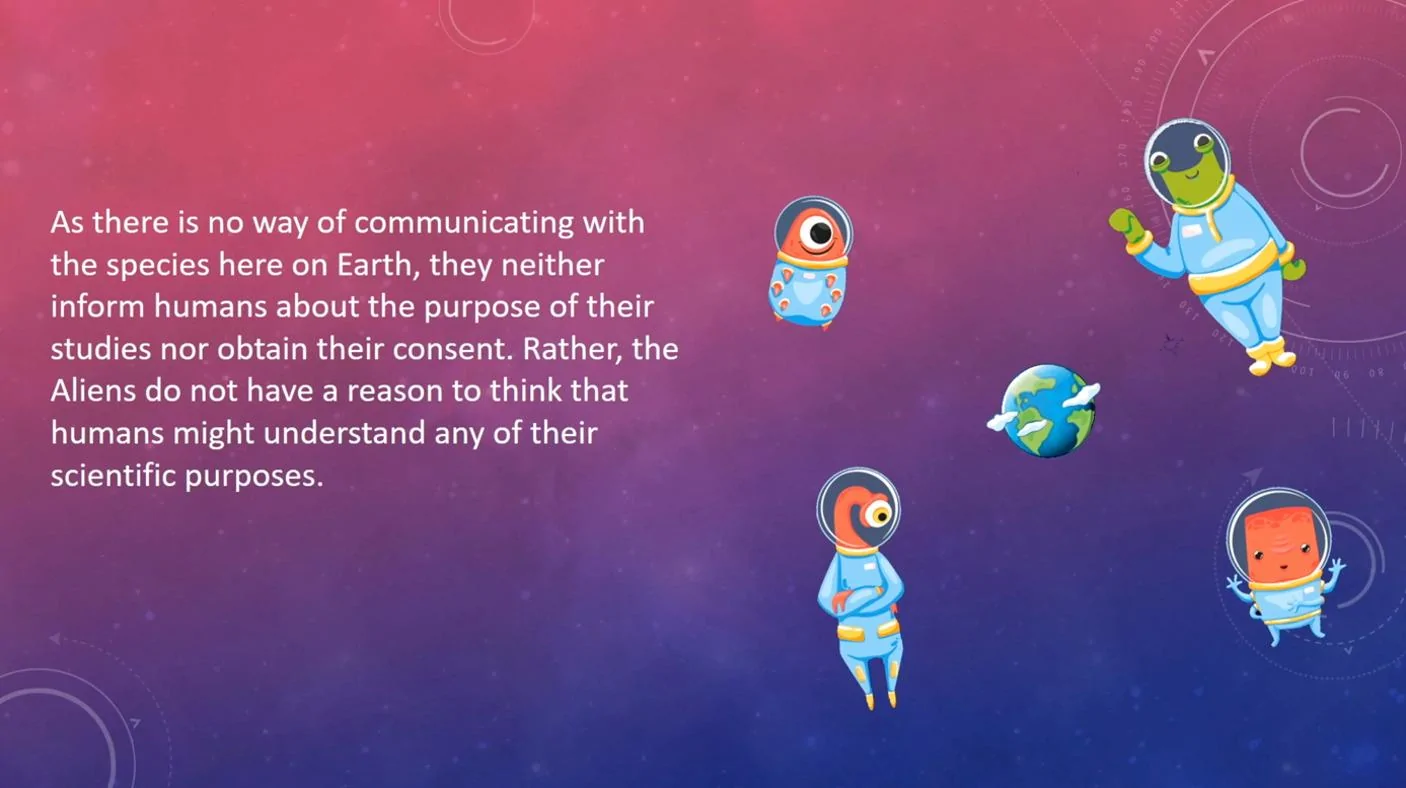
Snapshot of the Superior Scientist thought experiment video

Our experiment took the form of a video. Below is the audio transcript, with Fig. 1 providing a snapshot from the video.

*Imagine the following scenario*:

*An alien species called “the Aliens” is visiting Earth. They are quite special. Incredibly advanced in space travelling*,* they manage to overcome enormous distances in a very short time. That way*,* they are already familiar with quite a few planets*,* some of them even inhabited by life forms.*

*The Aliens are also impressively intelligent. They can communicate telepathically*,* or so it seems to humans. At least*,* humans don’t have a clue how the Aliens communicate with each other*,* but it must be very precisely*,* without exchanging words or gestures*,* as they collaborate very efficiently.*

*Furthermore*,* the Aliens are great scientists. They did not come to conquer Earth but they need to perform a number of experiments on the species living here to complete their knowledge about different life forms in space. The experiments involve behavioural studies*,* neurological studies*,* taking tissue and organ samples*,* enhancement studies*,* psychological experiments and much more.*

*As there is no way of communicating with the species here on Earth*,* they neither inform humans about the purpose of their studies nor obtain their consent. Rather*,* the Aliens do not have a reason to think that humans might understand any of their scientific purposes.*

*Surprisingly*,* the Aliens do have something in common with humans. In their research*,* they always follow the 3R. For Reduction*,* they take as few individuals as possible. For Refinement*,* they refine their methods to cause as little pain*,* stress*,* and harm as possible. For Replacement*,* they replace experiments on living individuals with those on dead individuals whenever possible. Also*,* they conduct the experiments on life forms who are less sentient*,* if possible.*

*However*,* for many of their study purposes*,* humans are necessary*,* as it is particularly the human life form that is of interest to them.*

*Do you think*,* the Aliens’ research is justified?*

*Do you think*,* the 3R are a good framework for their research?*

*Could you suggest alternative frameworks or guidelines?*

As we see, in our scenario aliens come to earth with what could be considered legitimate scientific aims: learning more about the universe and all life forms. The typical “consent approach” of human research is impossible in this case, both because humans cannot grasp the aims of the alien’s research and due to the impossibility to communicate. The aliens are described as great scientists, clearly more advanced than humans, which indicates that their research should at least be as suitable as that of humans. 3R is presented as a key element to justify their research. Finally, the thought experiment asks participants to reflect on whether the aliens’ research is justified, as well as on the extent to which 3R is the right framework for it.

### Study Design and Context

This study is part of the project “EXPLOR3R: Exploring 3R with Experimental Ethics”, funded by the National Research Program 79 “Advancing 3R” of the Swiss National Science Foundation. The aim of this project is to explore stakeholders’ perspectives and attitudes about 3R and animal research more generally, in the Swiss context.

### Participant Recruitment and Data Collection

Professionals in the field of animal research in Switzerland, such as researchers using animals, veterinarians, animal welfare officers, 3R counsellors, animal science trainers, and members of animal experimentation commissions, participated in the study (see Table 1 below). We recruited the participants using purposive sampling methods. Two authors (KP and CRP) searched for potential participants using institutional websites, both in the public sector (e.g., Swiss universities, Swiss 3R competence center) and the private sector (e.g., pharmaceutic industry). We ensured a representation of the diversity of roles in animal research, as well as in terms of age and experience of participants. Three authors (KP, CRP, and DS) sent emails to prospective participants in German, French, and English, depending on their profile and language area in Switzerland (Italian was not offered as an interview language due to the research team’s composition, but participants from the Italian-speaking part of Switzerland were not systematically excluded). They shared with participants an information document explaining the purpose of the project and study, its methodology, the potential risks and benefits, as well as a consent form. In total, 20 potential participants were contacted, of which 13 agreed to participate in an interview. In case of agreement, a date and time for the interview was scheduled. Before data collection, participants signed the consent form.

**Table 1:** List of participants with roles

Participant	Role
1	PhD Student – Biologist – Researcher
2	PhD Student – Molecular Biologist – Researcher
3	Research group leader – Biologist
4	Ecologist – Researcher
5	Animal Welfare Scientist
6	Research group leader – Molecular Biologist – Researcher
7	Animal Welfare Officer – Biologist
8	Animal Experimentation Commission member – Lawyer
9	3R Counsellor – Biologist
10	Animal Welfare Scientist – Veterinarian
11	Research group leader – Behavioral Biologist
12	Refinement researcher – 3R Counsellor - Biologist
13	Research group leader – Developmental Biologist

The methodology used for data collection was the semi-structured qualitative interview. For this purpose, a discussion guide was created before the interview. The guide included the participants’ journey in the field of animal research, their perspective on the 3R principles, their approach when it comes to applying 3R, their views on the Swiss animal research legal framework, on the killing of animals, as well as on the public perception of animal research and 3R, and the thought experiment described above. The guide was piloted with participants corresponding to our targeted profiles and was refined as we progressed. This paper focuses on the final section of the guide (i.e., the thought experiment). The thought experiment video was created by the authors (KP, CRP, and DS) using Microsoft PowerPoint Version 16.77, it is a series of slides with audio in German, French, or English. The video was displayed in the same language as the interview.

Three authors (KP, CRP, and DS) carried out the interviews (respectively) in German, French, and English, according to the participants’ preferences. The interviews took place either in person at a location agreed upon with the participants or online using the software Zoom with end-to-end encryption. The video was displayed on a computer during the in-person interviews and using the shared-screen function during the Zoom interviews. In two cases, two interviewers (KP and CRP) were present with one participant. In all other cases, only the interviewer and the participant were present. The interviews duration varied between 67 and 131 min long, the average being 92 min. All interviews were audio recorded with a recorder (in-person), or the Zoom recording function. The audio data were transcribed verbatim in the language of the interview using the software MAXQDA. In this process, anonymization was done by replacing all identifiable contents with neutral anonymous terms (e.g. “Participant 7”). All participants had the opportunity to check the transcripts. 3 participants asked to check the transcript and made minor changes in terms of content and anonymization. We ended data collection after data saturation was achieved.

### Data Analysis

Reflexive thematic analysis (Braun & Clarke, 2021) was used for data analysis. In a first step, two authors (KP and CRP) familiarized themselves with the data by reading a portion of the transcripts. In a second step, they independently thematically coded and generated themes based on the transcripts. The coding was both inductive and deductive, according to the exploratory hypothesis discussed above and the authors’ previous literature reviews. This second step also included a comparison of their results leading to a revision of the codes and themes. In a third step, the themes were renamed and restructured.

Data analysis for this paper is focused on the final section of the discussion guide, dedicated to the thought experiment. Different papers present the results of the remaining coding. To ensure that all data relevant to the thought experiment were taken into consideration, we thoroughly went through the paragraphs following the video till the end of the interviews.

**Fig. 2:**
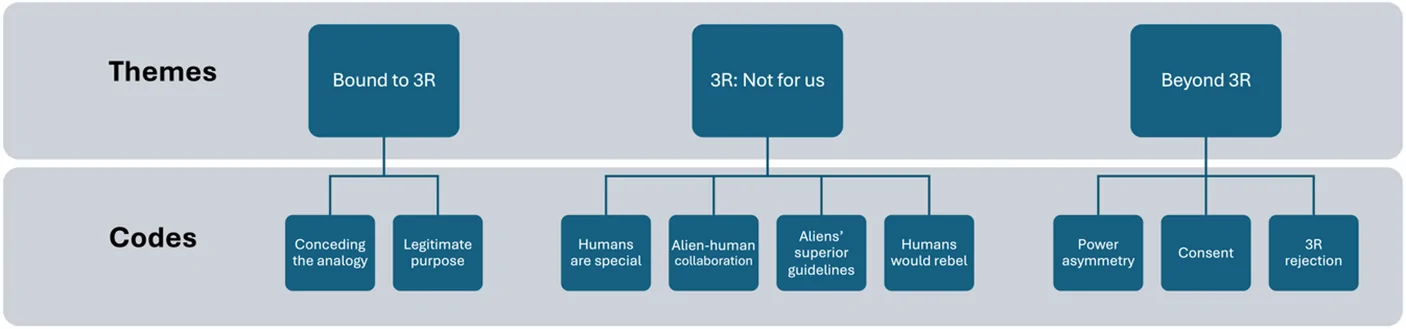
Resulting themes and codes

As Fig. 2 below shows, our thematic analysis resulted in 3 themes composed of a total of 9 codes.

Below, direct quotes from participants are used to support our findings. If not originally in English, these quotes were translated to English by two authors (KP and CRP), respectively fluent in German and French, and both fluent in English.

This study was conducted in line with the ethics approval received by the ethics committee of the University of Basel (UEK) on April 24th, 2023 (Reference number: 126).

## Results

The initial reactions to the Superior Scientist thought experiment were generally positive, often accompanied by laughter. However, despite the initial amusement, participants engaged seriously with the scenario, demonstrating a genuine willingness to reflect on its implications. This in itself is a significant result, as it suggests that the thought experiment was not only thought-provoking but also compelling enough to be taken seriously. Additionally, many participants expressed interest in using the scenario for various purposes, such as teaching.

Below, we present the resulting themes and codes using direct quotes from participants to support our findings.

### Bound to 3R

This theme reflects participants’ acceptance of 3R as a good framework for human research by aliens. The title was chosen to reflect the reluctant or ambivalent nature of this acceptance. Being bound to 3R, participants naturally referred to other components of animal research justification, such as legitimate purpose. This theme comprises two codes: “Conceding the analogy” and “Legitimate purpose”.

#### Conceding the Analogy

This code captures how, after discovering the thought experiment, most participants highlighted the analogy with phrases like “draw a parallel”, “pretty much the same”, “if the roles were reversed”, or “exactly like what we are doing”. We use the code “Conceding the analogy”, because many of them accepted the analogy with reluctance (e.g., “you have to admit”).

*And if I think that 3Rs are a good framework for their research. Yeah*,* well*,* sounds reasonable*,* right? This is exactly like what we’re doing with animals. So this is so interesting*,* these thought experiments*,* you know*,* because I think it really makes you think like*,* okay*,* it would not be okay for the aliens to experiment on humans. So why is it okay for us humans to experiment on animals*,* right? And then honestly*,* […] if you’re fine with […] animals*,* then you should not be outraged by propositions like this.* (Participant 4, pos. 705–711)

*It’s very difficult to accept*,* but from the point of view of aliens it’s completely justified*,* just as it is for us in regard to animals.* (Participant 10, pos. 86).

*Yes*,* of course*,* so from the aliens’ point of view we are justified. Yes. Probably*,* if you are intelligent*,* you have found something like 3 or 4 or even 5 Rs […] maybe they would just take organs from humans and study them*,* so the classic way of what human explorers do: take a specimen*,* cut it open*,* take it apart… a bright scenario*,* okay.* (Participant 11, pos. 3123–3124)

The quote below illustrates that, for participants who were positive about 3R in the context of animal research during the whole interview, it may have been challenging to criticize the principles when presented with the thought experiment.

*Do I think the 3Rs are a good framework for their research? Yeah*,* I think of all the arguments that I have presented so far*,* I would be in a bad place myself in terms of my coherence*,* if I wouldn’t think that they would be a good framework for the aliens*,* if I think they’re a good framework for us.* (Participant 5, pos. 567–568)

#### Legitimate Purpose

As previously stated, 3R is only one step towards the justification of animal research. A legitimate purpose for the experiment is another necessary condition. Many participants appealed to this requirement, as they were not totally satisfied with the legitimacy of the aliens’ research. One participant even drew a distinction between “curiosity-driven” research and translational research.

*If this research is just about discovering more on the [human] way of life and we have no evolutionary link with them*,* then maybe it’s less justified.* (Participant 1, pos. 592–593)

*There has to be a precise goal. Saying we want to improve knowledge is nice. But there still has to be an objective.* (Participant 9, pos. 124)

Other participants emphasized that the aliens’ research should benefit someone, or something, somewhere. They referred to the concept of harm-benefit analysis (HBA), which captures the requirement previously discussed (i.e., the expected benefits must outweigh the harms).

*If I follow my HBA question*,* if it would allow the aliens e.g.*,* their own species*,* but also ecosystems at the same time*,* they have already explored the whole galaxy*,* to improve something on this global level*,* then I would say that would be justified.* (Participant 13, pos. 618)

### 3R: Not for Us

This theme captures participants’ rejection of, or resistance to, the use of 3R for human research by aliens. Participants justified their objections in ways that led to the emergence of four codes: “Humans are special”, “Alien-human collaboration”, “Aliens’ superior guidelines”, and “Humans would rebel”.

#### Humans are Special

This code reflects the distinction often made by participants between humans and other animals used in research. This distinction was mostly presented to cast doubt on the justification of human use by aliens without compromising the justification of animal use by humans.

For example, humans have been presented as special for capacities such as their “intellectual faculties”, “sophistication”, and “self-awareness”.

*We are a lot more sophisticated life form. I don’t think directly the same 3Rs are applicable here somehow. But I cannot pinpoint it right now*,* other than being able to say we have a much more sophisticated life form.* (Participant 6, pos. 175)

Rather than appealing to capacities, some participants argued that humans are special because of “*the general international agreement we have for how to use humans”* or simply due to the fact of being human. Interestingly, the speciesist nature of the argument was directly noted.

*That’s nice of [the aliens] to do [3R]*,* but I’m already not okay with the basic assumption. Yes*,* because when I think about people*,* I have a completely different claim*,* namely that it is not justified*,* for whatever purpose*,* to somehow cause suffering in people. People who see it differently. And I’m clearly a speciesist*,* aren’t I? No matter how much I care about animal welfare and no matter how much I think that animal testing is problematic.* (Participant 12, pos. 837–852)

#### Alien-Human Collaboration

Despite the thought experiment explicitly presenting aliens as a superior species (e.g., impossibility for humans to communicate with them or grasp their scientific aims), some participants still elevated humans to the level of aliens. On that basis, a potential alien-human collaboration was suggested.

*In this metaphor*,* they are so much more advanced*,* maybe we could still find ways of communicating with them*,* so that we could share the scientific data we already have.* (Participant 1, pos. 600–603).

*If these aliens have a way of communicating with humans*,* then you would co-design studies*,* could you come up with some way of doing this that would be elaborated in accordance with those*,* the study population?* (Participant 5, pos. 571)

#### Aliens’ Superior Guidelines

It was surprising to some participants that the aliens, despite their superiority, could not develop guidelines that better accounted for differences between animals (including humans), for example by adapting the 3Rs. This expectation highlights the perceived limits of 3R for human research.

*At the same time*,* I think that if the aliens are so advanced*,* then they certainly have the means to conduct such research in the most human- or animal-friendly way possible. […] The crucial point for me is how the aliens assess the animals*,* i.e. the animals on Earth*,* and how they adapt the three R-rules accordingly. Do they treat humans in exactly the same way as an earthworm?* (Participant 13, pos. 576–585)

#### Humans Would Rebel

A few participants used a terminology of resistance and aggression to describe the scenario. The idea that humans would not accept to be used by aliens in this way also suggests that 3R is not for humans.

*But I don’t think we would be happy. And we would rebel. So that’s it*,* I don’t think we would just let it happen. […] but if we have no choice and we are anaesthetized by a remote laser device*,* then that’s it.* (Participant 3, pos. 188–192)

*Other than that*,* as a human being*,* obviously*,* I wouldn’t really appreciate being the object of an experiment.* (Participant 7, pos. 103)

### Beyond 3R

This third and last theme reflects how participants went beyond 3R, by pointing out its ethical flaws. The theme comprises three codes: “Power asymmetry”; “Consent”, and “3R rejection”.

#### Power Asymmetry

Participants often referred to the power asymmetry at play in the analogy. Rather than merely mentioning the use of one group by the other, they used terms such as “position of power”, “disturbing a species”, “have not chosen”, “kidnap”, or “forced to undertake experiments”. These attitudes were not limited to the Superior Scientist scenario, as they were also used to describe cases of animal research by humans, suggesting a more fundamental problem with 3R.

*It’s complicated because ultimately we’re in a position of power and maybe one day we won’t be*,* and maybe we’ll be the study project… and maybe they won’t have the 3Rs and we’ll suffer enormously.* (Participant 3, pos. 180)

*For [the aliens] it’s important*,* I’m sure they have their reasons and they can’t do otherwise. But at the same time you say why am I disturbing a species that was living its life*,* doing its thing*,* and I’m a superior being who chooses to say ‘Oh well*,* I want to do manipulations on you’.* (Participant 2, pos. 703–706)

#### Consent

We anticipated that consent, as a standard approach to research with humans, could play a role in participants’ attitudes, despite the fact that aliens would not necessarily care about human standards. Interestingly, consent was not only discussed within the context of the scenario, but also as something worth pursuing in animal research.

*It was described in the scenario that they did not ask for consent of the humans*,* right? It reminded me of*,* I think there’s discussion now*,* like in the animal ethics about like the animal consent*,* like in the animal experiments. And I think I was reminded of that*,* right? That even if you don’t speak the same language*,* or like how do you want to communicate with the animals*,* […] you can’t ask the animal or maybe like the aliens*,* not humans*,* […] do you want to participate? But you can still look for signs of consent or assent*,* […] or dissent*,* […] if you want to participate in research.* (Participant 4, pos. 690–705)

*And accordingly the alternative framework conditions and guidelines would be to work only with species or to work with individuals who can agree to this. […] Just like we would do would do with humans when we carry out in experiments.* (Participant 12, pos. 853–861)

#### 3R Rejection

It is worth mentioning that one participant was opposed to 3R in general, be it for research with humans or with animals. In their view, and in line with our previous discussion of absolute and relative dignity concepts, a better approach would be to respect the inherent value of both humans and animals.

*In the same way that we could propose other approaches or regulations for scientific research on humans*,* it would be to say ‘well no*,* don’t do this research if there is no strict respect for the dignity of the individuals [including animals] on whom we are experimenting’.* (Participant 8, pos. 111)

## Discussion

The results support our initial hypotheses by revealing a diversity of attitudes, ranging from acceptance to rejection of 3R for human research by aliens. As we expected, appeals to other relevant concepts beyond 3R have been made. Additionally, unexpected insights emerged, including the role of animal consent and the concept of alien-human collaboration.

A key finding is that all participants engaged in the discussion and evaluation of the analogy without rejecting it outright. The Superior Scientist encouraged participants to consider Firestone’s key question regarding such analogies: “What principles of justice would we choose to govern the interactions between species if we were not the most intelligent and powerful species on earth?” (Firestone, 2016). This reflects the Rawlsian idea of asking whether we would still endorse the same societal rules if our roles were different, or instead propose fairer rules for all members of society. Several quotes relating to power asymmetry and human rebellion highlight how participants conceptualized the imbalance in these relationships.

Nonetheless, some participants remained “Bound to 3R” and engaged with the analogy using a typical 3R approach (e.g., how to refine experiments for human subjects). Therefore, it was not surprising to see legitimacy emerge as a major topic. Participants questioned whether the research conducted by aliens had a justifiable purpose, mirroring common concerns raised in discussions on animal research. By distinguishing between “curiosity-driven” and translational research, participants applied familiar concepts and created a parallel with an ongoing debate in animal research (Eggel et al., 2019).

Other participants rejected 3R for humans, as reflected in the “3R: Not for us” theme. There, arguments tended to define humans as special compared to other animals, either based on capacities (e.g., sophistication) or on intrinsic value (e.g., being “people”). This confirms our prediction that species-specific characteristics, as well as anthropocentrism and speciesism, would play a role in participants’ attitudes. Accordingly, aliens’ reliance on 3R was considered limited and more advanced considerations of species differences were hoped for. As anticipated, there were cases in which participants tried to justify their initial intuitions through rationalization, as is evident in quotes such as “*I don’t think directly the same 3Rs are applicable here somehow. But I cannot pinpoint it right now […]*”. From this perspective, arguments related to alien-human collaboration or aliens’ superior guidelines can be seen as attempts to reconcile participants’ intuition. For example, by elevating humans to the level of aliens as “fellow scientists”, participants overlooked key constraints of the scenario and could thereby rationalize the view that applying 3R to humans was inappropriate. Alternatively, this line of reasoning may suggest that the experiment did not function entirely as intended, and that participants remained anchored in their scientists’ perspective (i.e., aligned with the aliens), rather than seriously considering the possibility of becoming research subjects.

Some participants’ attitudes went directly “Beyond 3R”, with consent as a critical factor. While we anticipated that the standard requirement of consent in human research would play a role, an unexpected finding was the discussion of consent by animals. In line with current discussions in animal research ethics (Mancini & Nannoni, 2022), some participants suggested that animal assent or dissent could provide an ethically superior approach to 3R, reasoning that research should be limited to individuals capable of giving consent. The issue of consent is also connected with the underlying power asymmetry, which was also highlighted by participants and further challenges the adequacy of 3R. Finally, only one participant radically rejected 3R, questioning whether any form of non-consented research could be justified and suggesting an absolute dignity approach for both humans and animals.

A limitation of our study lies in the fact that participants were presented with the thought experiment at the very end of the interview. One could expect that discussing 3R during the whole interview would have biased participants’ attitudes towards these principles. In particular, some participants were positive about 3R in the context of animal research which may have made it challenging for them to criticize the principles when applied to humans by aliens. It could be said that the justification for 3R in this experiment was often circular, or at least tautological (i.e., 3R is a good framework (here) because we must believe it is a good framework (in animal research)). Fatigue and time-constraint are also worth noting, since some interviews exceeded the planned 120 min. As a result, participants may have shortened their answers to conclude the interview more quickly. Finally, a potential social desirability bias must be mentioned. 3R is often considered an ethical framework, and discussing this topic in interviews conducted by members of an ethics institute could create an environment where participants feel compelled to align their responses with perceived ethical norms or expectations.

Nonetheless, our findings indicate that the thought experiment provoked ethical reflection and provided valuable insights into participants’ attitudes to 3R and animal research ethics. The results suggest that 3R in human research (by aliens) was not seen as an adequate framework, given the ambivalent acceptance by some participants, the outright rejection by others (sometimes relying on gut feeling), and the calls to look beyond 3R. If the analogy holds, this finding also challenges the adequacy of 3R in animal research (by humans), supporting previously identified concerns about their limitations. Participants’ concerns about legitimacy, consent, power asymmetry, and limitations of 3R, underscores the ongoing need to advance ethical frameworks in animal research.

Our approach and results have attracted interest from a diverse range of scholars and educators, highlighting their potential for integration into the training of future researchers. We hope that one lasting impact of this work will be to foster broader adoption of experimental ethics methods and critical ethical reflection as a core component of scientific education. Future research could expand on our findings by investigating the solutions proposed by participants, such as ethical approaches grounded in animal consent or human-animal collaboration, and examining how professionals envision implementing them. It would also be valuable to test the consistency of these attitudes across different populations, including laypeople, policymakers, and scientists from other fields, as well as across cultural contexts.

## Conclusion

The Superior Scientist thought experiment challenged participants’ intuitions about the justification of animal research and the role of 3R, resulting in a spectrum of attitudes toward the application of 3R to humans. Thematic analysis identified three patterns: ambivalent acceptance, outright rejection (sometimes relying on gut feeling), and calls to look beyond 3R. The results highlight the importance of aspect such as species-specific capacities, anthropocentrism, speciesism, consent, power asymmetry, and the purpose of research, as well as the overlooked potential of animal consent and interspecies collaboration. These findings support previously identified concerns about the limitations of 3R and show that experts also recognize these challenges when reflecting on the issue using a role-reversal thought experiment. This underscores the ongoing need to advance ethical frameworks in animal research, expanding beyond 3R to incorporate broader moral considerations. Ultimately, our findings suggest that empirical animal ethics methods, in particular thought experiments of this kind, offer a promising route to fostering ethical reflection and reform.

## Data Availability

Anonymized transcripts are accessible upon request at the Institute for Biomedical Ethics, University of Basel.
